# Advancing the Capability of Additively Manufactured Continuous Fibre-Reinforced Polymers for Structural Applications: The Effect of Nitrogen-Purging and Post-Annealing on the Tensile Performance

**DOI:** 10.3390/polym17172314

**Published:** 2025-08-27

**Authors:** Zizhao Peng, Jiahui Li, Yvonne Durandet, Antonella Sola, Adrian Trinchi, Phuong Tran, Wei Gao, Xuemei Liu, Dong Ruan

**Affiliations:** 1 Department of Mechanical and Product Design Engineering, School of Engineering, Swinburne University of Technology, Hawthorn, VIC 3122, Australia; zpeng@swin.edu.au (Z.P.); jiahuili@swin.edu.au (J.L.); ydurandet@swin.edu.au (Y.D.); 2 Victorian Hydrogen Hub (VH2), Swinburne University of Technology, Hawthorn, VIC 3122, Australia; 3 Manufacturing Research Unit, The Commonwealth Scientific and Industrial Research Organisation (CSIRO), Clayton, VIC 3168, Australia; adrian.trinchi@csiro.au; 4 Department of Sciences and Methods for Engineering, University of Modena and Reggio Emilia, 42122 Reggio Emilia, Italy; 5 RMIT Centre for Additive Manufacturing, RMIT University, Melbourne, VIC 3000, Australia; jonathan.tran@rmit.edu.au; 6 Centre for Infrastructure Engineering and Safety (CIES), School of Civil and Environmental Engineering, The University of New South Wales, Sydney, NSW 2052, Australia; w.gao@unsw.edu.au; 7 Department of Infrastructure Engineering, Faculty of Engineering and Information Technology, The University of Melbourne, Parkville, VIC 3052, Australia; xuemei.liu@unimelb.edu.au

**Keywords:** additive manufacturing, material extrusion, continuous fibre-reinforced polymers, composites, mechanical properties, nitrogen-purging, post-annealing

## Abstract

Additively manufactured continuous fibre-reinforced polymers (CFRPs) offer promising mechanical properties for engineering applications, including aerospace and automotive load-bearing structures. However, challenges such as weak interlayer bonding and low strength compared to traditional composites remain. This paper presents an experimental investigation into the effects of nitrogen (N_2_) purging during printing and thermal annealing after printing on the tensile performance of additively manufactured CFRPs. Tensile tests were conducted on Onyx specimens produced by material extrusion and reinforced with continuous carbon fibre filaments (CFF), glass fibre filaments (GFF), or Kevlar fibre filaments (KFF). Results showed that N_2_-purging and post-annealing had different effects on the tensile properties of various CFRPs. Particularly, N_2_-purging, post-annealing, and their combination enhanced both the Young’s modulus and ultimate tensile strength (UTS) of KFF/Onyx specimens. For GFF/Onyx specimens, both treatments had a minor effect on the Young’s modulus but enhanced UTS. CFF/Onyx specimens exhibited improved Young’s modulus with N_2_-purging, while both treatments reduced UTS. The different response of the CFRPs was associated with diverse governing failure mechanisms, as proved by microstructural and fracture surface inspection. Additionally, differential scanning calorimetry (DSC) and X-ray diffraction (XRD) analyses also revealed the thermal behaviour and crystal structures that influence the mechanical properties of CFRPs.

## 1. Introduction

Recently, there has been an increasing interest from various industries in utilising additive manufacturing (AM) for rapid prototyping and advanced engineering applications [1,2]. The Wohlers Report 2023 [3] highlights several applications of AM in the aerospace and automotive industries. In aerospace, Lufthansa has applied AM polymer air ducts and fasteners within aircraft cabins. In the automotive sector, BMW has incorporated AM components in its iX5 hydrogen vehicle, such as the front grill cover, air inlets, and rear trim. NASCAR has also employed AM to improve its race car performance, using a Stratasys H350 machine to print cockpit ventilation units for its Next Gen cars. AM enables low-quantity and high-quality fabrication with minimal material wastage [4]. It provides a fast and ideal approach for fabricating a small volume of newly designed, custom-made, or spare parts with complex geometries, which are expensive and time-consuming to produce using traditional manufacturing methods [5,6]. Moreover, the geometry of a part can be easily modified for AM to obtain an optimal topology that possesses desired mechanical properties with minimum weight [7,8]. Among the various AM techniques available, Material Extrusion (MEX), especially Fused Deposition Modelling (FDM), has gained widespread popularity due to its user-friendly nature and capability to print an extensive variety of materials [9,10]. MEX-printed continuous fibre-reinforced polymers (CFRPs) offer significant practical advantages in industrial applications, primarily owing to their competitive mechanical properties and lightweight [11]. They demonstrate great potential to be alternatives to metal components [12].

Previous research has been focused on the analysis of the mechanical performance of MEX-printed polymers such as acrylonitrile butadiene styrene (ABS), polylactic acid (PLA), Nylon (i.e., polyamide), Polyether ether ketone (PEEK), and their corresponding short and continuous fibre-reinforced composites [13]. However, due to the hygroscopic nature of matrix materials, printed CFRPs absorb moisture from the atmosphere quickly. This absorbed moisture negatively impacts mechanical properties by inducing plasticisation effects and creating pores within the deposited strands of material (“rasters”), thereby compromising structural integrity [14,15]. Further to this, the poor interlayer adhesion between the fibre-reinforced layers and the matrix ones caused by residual thermal stresses always remains a serious issue, impairing the structural strength and reliability of nowadays MEX-printed structures [16]. It is thus imperative to enhance the mechanical performance of MEX-printed CFRPs, particularly for applications where the demand for lightweight, high-strength materials is critical.

Improving the printing conditions or applying post-printing treatments [17,18,19] are two options to ameliorate the performance of MEX-printed parts. Regarding the printing conditions, most MEX products are routinely printed under normal atmospheric conditions. As a result, the feedstock filament is likely to absorb moisture and various gases, such as oxygen, which may affect the printing quality and the performance of the products. To provide an inactive and dry printing environment, purging with inert gases such as N_2_ and argon (Ar) into the chamber of the printer has been taken into consideration. This may also improve the layer consolidation throughout the MEX printing process and lead to high-quality products. For example, Shaik et al. [20] printed PLA specimens using MEX under compressed air and in N_2_ atmosphere. Their results showed that N_2_-purging increased the Young’s modulus of MEX-printed PLA specimens by 30% and 50% in the longitudinal and transverse orientations, respectively, and improved the yield strength by 40% in both orientations, achieving mechanical properties comparable to those of injection-moulded specimens.

Post-annealing is a post-processing method that favours the mechanical performance of the printed parts by eliminating the residual moisture and by obtaining better fusion between adjacent rasters and layers [21,22]. In addition, polymers may also undergo structural changes. For instance, post-annealing enables molecular chain reorganisations and promotes crystallisation [23]. The optimal post-annealing temperature to achieve the best mechanical performance is material-specific and should be determined on a case-by-case basis [24,25,26].

Handwerker et al. [27] investigated the impact of annealing on polyamide 6 (PA6) composites reinforced with continuous glass fibres (cGF) and short carbon fibres (sCF), manufactured via a Markforged^®^ Mark Two printer (Markforged Inc., Watertown, MA, USA). Annealing doubled the Young’s modulus and increased UTS by 50% in sCF/PA6 composites, while cGF/PA6 specimens demonstrated a 186% UTS increase with a crystallinity rise from 23% to 27%. Optimal annealing conditions for both materials were identified as 200 °C for six hours. Moreover, Wang et al. [28] reported that post-annealing of continuous carbon fibre-reinforced PEEK (cCF/PEEK) composites at 250 °C for six hours enhanced crystallinity, interlayer bonding, and UTS by 16%, with the interlaminar shear strength increasing by 85%. However, annealing at 300 °C led to mechanical degradation. In addition, Muna et al. [29] compared two post-annealing methods for continuous carbon fibre-reinforced PLA composites (cCF/PLA): constant annealing at 65 °C and cyclic annealing between 50 °C and 70 °C, both for six hours. Both methods reduced the Young’s modulus and UTS of cCF/PLA, with constant annealing showing decreases of 11% and 3.6%, respectively, and cyclic annealing causing decreases of 28% and 9.6%, respectively.

The literature suggests that both N_2_-purging and post-annealing can have significant effects on the printing quality and mechanical properties of AM materials such as PLA, sCF/PA6, cGF/PA6, and cCF/PEEK. As indicated by previous studies, N_2_-purging has improved the mechanical properties of MEX-printed PLA by mitigating the adverse effects of moisture uptake and oxidation during the printing process [20]. However, the effect of N_2_-purging on the mechanical performance of AM CFRPs is not clear yet. As a cost-effective and environmentally sustainable technique, N_2_ is readily available from the atmosphere and can be efficiently recycled from exhaust air. Furthermore, post-annealing has improved the mechanical performance of MEX-printed parts such as sCF/PA6, cGF/PA6 [27], and cCF/PEEK [28] by enhancing fibre-matrix bonding and reducing the moisture content. However, systematic investigations of the effects of N_2_ purging and post-annealing on diverse AM CFRPs remain scarce. Particularly, no research has investigated the effect of a combination of N_2_-purging and post-annealing on the mechanical performance of AM CFRPs. Given the unsatisfactory mechanical performance of AM CFRPs, there is a critical need to develop effective processing or post-processing strategies that can enhance the mechanical performance of AM CFRPs. Therefore, the primary objective of the present study is to explore whether the tensile properties of three distinct types of CFRPs, i.e., carbon fibre filament reinforced Onyx (CFF/Onyx), glass fibre filament reinforced Onyx (GFF/Onyx), and Kevlar fibre filament reinforced Onyx (KFF/Onyx), are affected by N_2_-purging upon printing or post-annealing after printing, or a combination of both treatments.

In this study, tensile tests were first conducted on CFF/Onyx, GFF/Onyx, and KFF/Onyx specimens under various printing and post-processing scenarios. Microstructural observations were also carried out using scanning electron microscopy (SEM) to study the dominant modes of failure, identify the changes triggered by post-annealing at the microscopic scale, and gain an in-depth understanding of the tensile test results and failure mechanisms. Furthermore, differential scanning calorimetry (DSC) and X-ray diffraction (XRD) analyses were performed to unveil phase changes within the polyamide (PA) matrix of Onyx, variations in crystallinity, and fluctuations in moisture content. Such transformations have been considered as critical factors affecting the mechanical characteristics of the CFRPs.

## 2. Materials and Methods

### 2.1. Printer and Materials

All CFRP specimens were produced using a Markforged^®^ desktop 3D printer, Mark Two Generation II (Markforged Inc., Watertown, MA, USA), and the slicing software Eiger (software version as of 1 March 2023). Mark Two is a dual-extrusion printer with two individual extruding nozzles, which enable the Onyx and fibre filament to be deposited independently [30] and obtain different fibre filament volume fractions. In this study, the fibre filament volume fraction (Vf) refers to the proportion between the consumed fibre filament (polymer impregnated continuous fibre) and the total consumed materials, including the matrix filament (Onyx) and fibre filament. Vf is automatically calculated in Eiger. The two nozzles can also ensure better printing quality by minimising the inconsistent distribution of fibres that is commonly observed in co-extrusion printing [31,32,33].

Onyx filament (which is made of micro carbon fibre-filled nylon/PA [34]) supplied by Markforged^®^ (Markforged Inc., Watertown, MA, USA) with a diameter of 1.75 mm was employed as the matrix material for all the CFRPs. To avoid any moisture absorption and related deterioration before printing, Onyx was stored in a dry box. Markforged^®^ continuous carbon, glass, and Kevlar fibre filaments (KFF) were used as reinforcements in this study. The properties of the Onyx and fibre filaments retrieved from Markforged^®^ datasheets [35] are summarised in Table 1.

### 2.2. N_2_-Purging

In experimental groups designed to investigate the effect of N_2_-purging, the printer was purged with pure and dry N_2_ at room temperature at a flow rate of 8 L/min throughout the printing process. To maintain a controlled air pressure during printing, the printer was sealed with sticky tape, leaving necessary minor gaps for ventilation. The chamber temperature (CT) and relative humidity (RH) were measured using INKBIRD IBS-TH2 sensors (INKBIRD Tech. Co., Ltd., Shenzhen, China) located inside the printing chamber and recorded at a 0.1 Hz logging rate. The sensors showed that the change in CT during printing, both with and without N_2_-purging, was negligible, while the average RH was reduced from 27.9% to 6.4% by applying N_2_-purging. Detailed CT and RH data for all the specimens are summarised in Appendix A.

### 2.3. Post-Annealing

Annealing of CFRP specimens after printing was conducted in a thermostat fan-forced heating oven (Model MRX-GF50L, Mingruixiang Automation Equipment Co., Ltd., Shenzhen, China). Specimens were annealed for two hours, then removed from the oven and cooled to room temperature. The annealing temperatures were selected above the glass transition temperature (Tg = 64.5 °C [36]) of the Onyx matrix material. Preliminary uniaxial tensile tests were conducted on GFF/Onyx specimens after being annealed at temperatures of 90 °C, 120 °C, 150 °C, 180 °C, and 210 °C for two hours. Results revealed minimal mechanical property differences between specimens treated at 90 °C and 120 °C. Specimens annealed at 150 °C exhibited the highest Young’s modulus and UTS, whilst specimens melted after annealing at 210 °C. Consequently, annealing temperatures of 90 °C, 150 °C, and 180 °C were selected for the main study.

In addition, preliminary tensile tests were performed to determine the optimal post-annealing duration. Based on five indicators, including Young’s modulus, UTS, failure strain, strain energy, and annealing duration/energy consumption, specimens post-annealed for two hours demonstrated the most favourable outcomes. A detailed evaluation of post-annealing duration is provided in Appendix B.

### 2.4. Mechanical Characterisation

#### 2.4.1. Feedstock Fibre Filaments

Before testing the printed CFRP composite specimens, it was deemed useful to investigate the effects of annealing on the feedstock fibre filaments in order to provide a benchmark. For this purpose, 200 mm long segments of feedstock carbon fibre, glass fibre, and KFF were cut from the spools supplied from Markforged^®^ and annealed at 150 °C for two hours. Uniaxial tensile tests were conducted on as-received and annealed CFF, GFF, and KFF using a universal testing machine (Instron Model 5965, Instron, Norwood, MA, USA) equipped with a 10 kN load cell. Since all the feedstock fibre filaments are thin and fragile, the loading speed was set at 0.5 mm/min (strain rate of 5.6 × 10^−5^/s). To ensure that the filaments were gripped firmly, each end of each specimen was fixed and sandwiched between two thermoplastic polyurethane (TPU) tabs. Three specimens were tested for each condition.

#### 2.4.2. Printed Onyx and CFRP Specimens

To evaluate the effects of N_2_-purging, post-annealing, and their combination on the printed Onyx, neat Onyx specimens were printed following the Type I specification outlined in the ASTM D638 standard [37]. The printing raster angle was ±45°. In this case, three groups of specimens were examined and categorised as “AP”, “N_2_”, and “N2+FO150”, representing the as-printed, N_2_-purged only, and N_2_-purged with additional fan oven post-annealing at 150 °C, respectively. A universal test machine, MTS Model 43 (MTS System Corporation, Eden Prairie, MN, USA), with a load cell of 50 kN, was operated at 2 mm/min (strain rate of 6.7 × 10^−4^/s) for these tests.

**Figure 1:**
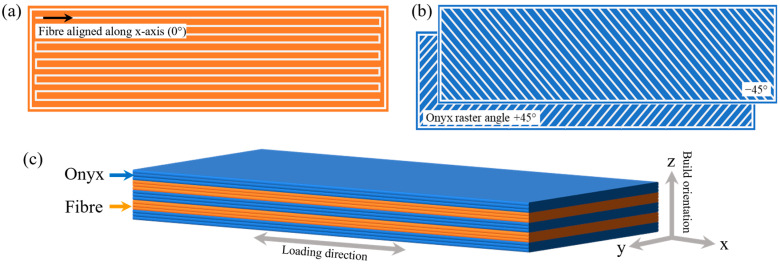
Schematic diagrams of the raster angles for (**a**) fibre-reinforced layers (0°); (**b**) Onyx layers (±45°); and (**c**) 3D schematic diagram of group-alternating stacking sequence with build orientation and tensile loading direction indicated. Note: dimensions are not to scale.

All CFRP tensile specimens were printed on an unheated printing bed with a solid infill pattern and 100% infill density. In accordance with ASTM D3039 standard [38], the dimensions for the tensile composite specimens were 250 × 25 × 2.5 mm^3^. To take full advantage of the reinforcing effect of the continuous fibre filaments, the fibre filaments were printed at a 0° angle along the longitudinal direction of the specimen, as depicted in Figure 1a. The Onyx layers were printed with an alternating raster arrangement (±45°) as shown in Figure 1b. In addition, a group-alternating stacking sequence was employed for all the specimens, as illustrated in Figure 1c, where the blue and orange layers represent the Onyx and the fibre-reinforced layers, respectively.

The layer thickness was 0.125 mm for both fibre-reinforced and Onyx layers in CFF/Onyx specimens, and 0.1 mm for both fibre-reinforced and Onyx layers in both GFF/Onyx and KFF/Onyx specimens. For each layer, two walls of Onyx were printed at the perimeter. The nozzle temperatures for the Onyx and fibre filaments were 275 °C and 255 °C, respectively [39]. The fibre filament Vf was kept constant for the three different CFRPs (with Vf designed to be 34%) as the primary aim of this study was to assess the effect of printing atmosphere and post-annealing by comparing the mechanical properties of CFRPs under uniform Vf conditions to isolate the effect of fibre type. To confirm, the slicing software calculated that the fibre filament Vf was 33.72%, 34.14% and 34.56% for the CFF/Onyx, GFF/Onyx, and KFF/Onyx specimens, respectively. The specific configuration for each CFRP type is summarised in Table 2.

Six distinct test groups were analysed for each type of CFRP: Group 1 contained as-printed specimens, denoted as “AP”, which were printed using the default printing setting without N_2_-purging nor post-processing; Group 2 contained specimens printed with N_2_-purging (N_2_); Groups 3–5 were specimens post-annealed in a fan oven at 90 °C (FO90, Group 3), 150 °C (FO150, Group 4) and 180 °C (FO180, Group 5); Group 6 gathered specimens printed with N_2_-purging and post-annealing at 150 °C (N2+FO150). For Groups 3–5, the dimensions and weights of all specimens were measured before and after post-annealing.

Tensile tests were performed using an MTS Model 43 universal machine with a 50 kN load cell. In accordance with the ASTM D3039 standard [38], the test speed was set to 2 mm/min (strain rate of 6.7 × 10^−4^/s). Three specimens were printed and tested for each group. Each specimen had a gripping length of 50 mm at both ends, and a video extensometer was employed to ensure accurate strain measurement.

### 2.5. Microstructural Analysis

The fracture surfaces of tensile-tested specimens were analysed using a Gemini 360 scanning electron microscope (Carl Zeiss Microscopy GmbH, Oberkochen, Germany) to investigate the failure mechanisms and morphological interfacial characterisation of neat Onyx and CFF/Onyx under AP and FO150 conditions. In addition, the effects of post-annealing at 180 °C for two hours were examined on CFF/Onyx and KFF/Onyx specimens to further explore microstructural changes and fibre-matrix interactions subjected to post-annealing at a higher temperature. All SEM observations were performed on standard tensile specimens to ensure consistency with mechanical testing conditions.

### 2.6. Thermal Characterisation

DSC analyses were performed using a TA Instrument DSC Q1000 model (TA Instruments, New Castle, DE, USA) on the as-printed and post-annealed Onyx, CFF/Onyx, GFF/Onyx, and KFF/Onyx specimens. For each specimen, two heating runs were performed from 25 °C to 300 °C at a heating rate of 25 °C/min with intermediate cooling at the same rate back to 25 °C.

### 2.7. X-Ray Diffraction

To complete the DSC analysis and confirm the phase composition of Onyx and CFRP specimens, XRD analysis was performed using a Bruker D2 Phaser benchtop instrument (Bruker AXS GmbH, Karlsruhe, Germany) at 30 kV with a cobalt X-ray tube (a wavelength of 1.79026 Å). The same neat Onyx and CFRP specimens printed for tensile testing were also used for XRD.

## 3. Results and Discussion

### 3.1. Tensile Testing

#### 3.1.1. Tensile Properties of Feedstock Fibre Filaments

Tensile test results of the feedstock fibre filaments are summarised in Table 3. Post-annealing at 150 °C has a negligible impact on the Young’s modulus of CFF, but a detrimental effect on both its UTS and failure strain. Conversely, the Young’s modulus, UTS, and failure strain of GFF and KFF are all improved after post-annealing. The effect on UTS is especially significant, with values that nearly double after post-annealing.

#### 3.1.2. Tensile Properties of Neat Onyx Specimens

**Figure 2:**
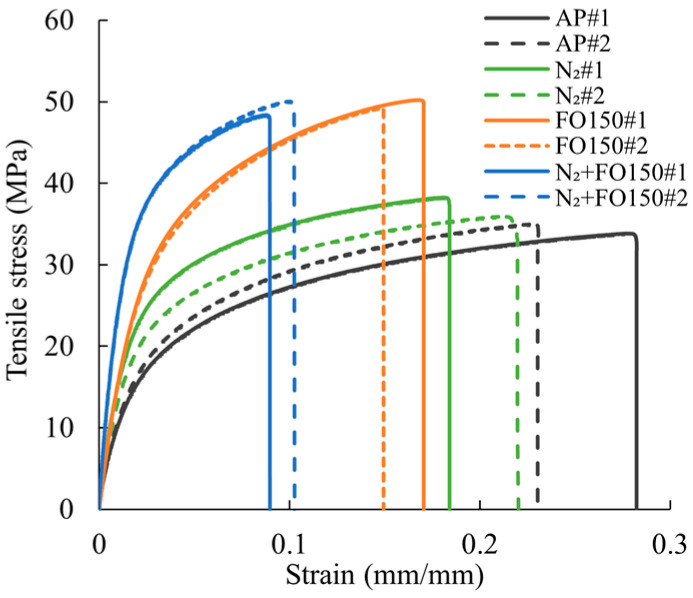
Stress-strain curves of neat Onyx specimens under various scenarios.

Figure 2 illustrates the stress-strain curves of the neat Onyx specimens in four groups: AP, N_2_, FO150, and N2+FO150. The Young’s modulus, UTS, and failure strain of the neat Onyx specimens are summarised in Table 4. In comparison to the AP specimens, the N_2_ specimens show a 43% increase in Young’s modulus, accompanied by a modest 9% increase in UTS. The FO150 specimens exhibit more substantial enhancements, with a 59% increase in Young’s modulus and a 47% increase in UTS. For the N2+FO150 specimens, a dramatic improvement (174%) in the Young’s modulus is observed, while UTS also increases by 44%. However, the failure strain for N_2_, FO150, and N2+FO150 specimens decreases compared to the AP specimens. This reduction indicates that both N_2_-purging and post-annealing have a negative impact on the ductility of neat Onyx specimens.

#### 3.1.3. Tensile Properties of CFRP Specimens

The detailed comparison of Young’s modulus, UTS, and failure strain of the different CFRPs is listed in Table 5. The specific effects of purging, annealing, and their combination are discussed below in the dedicated sub-sections.

##### N_2_-Purged CFRP Specimens

**Figure 3:**
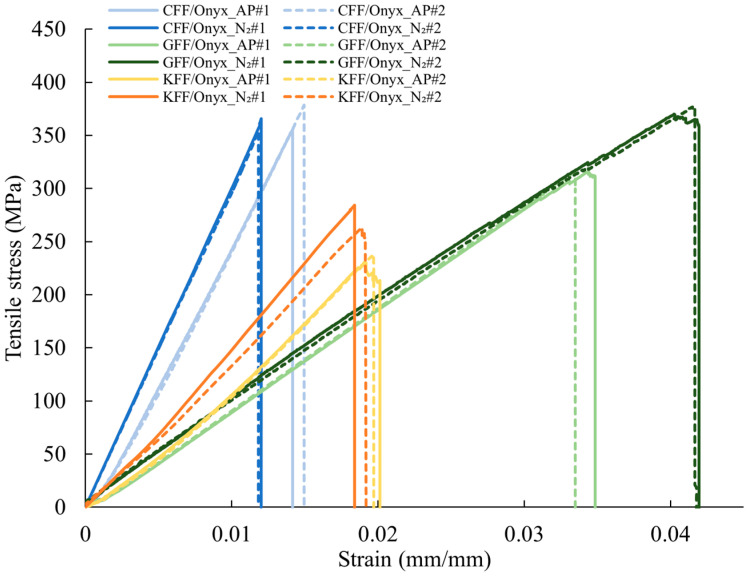
Stress-strain curves of the CFF/Onyx, GFF/Onyx, and KFF/Onyx specimens under both AP and N_2_ conditions.

The stress-strain curves of the CFRP specimens under both AP and N_2_-purged conditions are presented in Figure 3. For all the specimens, the stress increases linearly with strain till the UTS is reached, when brittle failure occurs. The failure strain is the highest for GFF/Onyx and the lowest for CFF/Onyx under both AP and N_2_-purged conditions. The effects of N_2_-purging on the failure strain of all CFRPs are negligible (less than approximately 1%). N_2_-purging results in significant improvements in the Young’s modulus for the CFF/Onyx and KFF/Onyx specimens. However, the GFF/Onyx specimens subjected to N_2_-purging experience a minor change in Young’s modulus.

##### Post-Annealed CFRP Specimens

**Figure 4:**
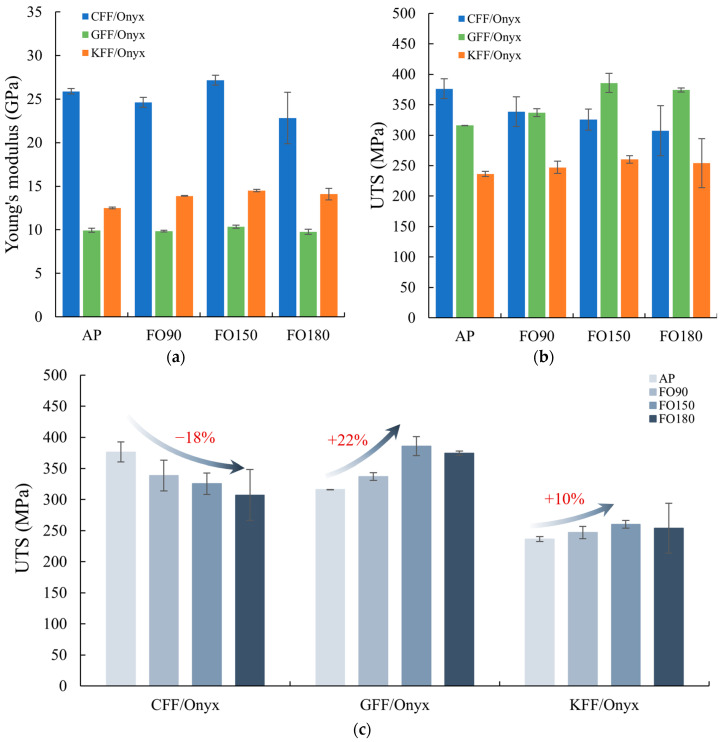
(**a**) Young’s modulus; (**b**) UTS of CFF/Onyx, GFF/Onyx, and KFF/Onyx composites under different fan oven annealing temperatures; (**c**) trends of UTS for CFF/Onyx, GFF/Onyx, and KFF/Onyx composites.

Figure 4 compares the Young’s modulus and UTS of CFF/Onyx, GFF/Onyx, and KFF/Onyx CFRPs subjected to post-annealing at various temperatures, with respect to AP specimens. According to Figure 4a, among all the CRFCs annealed at the same temperature, CFF/Onyx specimens exhibit the highest Young’s modulus. For CFF/Onyx, the Young’s modulus remains similar after post-annealing at temperatures of 90 °C and 150 °C, but decreases after annealing at 180 °C due to the prolonged exposure to high temperatures. GFF/Onyx specimens exhibit a negligible change in Young’s modulus after annealing at all temperatures, while KFF/Onyx specimens show a notable increase in Young’s modulus at 150 °C. As shown in Figure 4b, post-annealing progressively reduces the average UTS of CFF/Onyx as the annealing temperature rises to 180 °C. In contrast, after annealing at 150 °C, GFF/Onyx specimens experience a considerable improvement in UTS, whereas the KFF/Onyx specimens exhibit a smaller increase. As indicated by the error bars shown in Figure 4a,b, the data is less scattered for both GFF/Onyx and KFF/Onyx than for CFF/Onyx. Significant reduction and instability appear in both Young’s modulus and UTS of CFF/Onyx specimens when the temperature reaches 180 °C due to carbon fibre embrittlement and extensive thermal expansion after post-annealing. This phenomenon will be discussed in Section 3.2.1.

As observed in Figure 4c, there exist clear relationships between UTS and annealing temperature. The UTS of CFF/Onyx decreases monotonically with the annealing temperature, while the UTS of GFF/Onyx and KFF/Onyx peaks at 150 °C. It is noteworthy that the UTS of the GFF/Onyx_AP specimens is, on average 16% lower than that of the CFF/Onyx_AP. However, the UTS of GFF/Onyx_FO150 remarkably improved to an average of 386 MPa, surpassing that of CFF/Onyx_AP by 2.7%. Compared to CFF/Onyx and GFF/Onyx, post-annealing demonstrates a smaller impact on the UTS of KFF/Onyx.

##### Combination of N_2_-Purging and Post-Annealing

Because the optimal annealing temperature is 150 °C for both GFF/Onyx and KFF/Onyx specimens, one extra set of specimens was printed in a N_2_-purged environment and subsequently post-annealed at 150 °C for two hours (N2+FO150). According to the test results, when subjected to the combined treatment, GFF/Onyx specimens exhibit the largest failure strain (0.044), whereas CFF/Onyx specimens show the smallest failure strain (0.011). For both CFF/Onyx and GFF/Onyx, there is almost no change in the Young’s modulus between N_2_ and N2+FO150, whereas for KFF/Onyx, an increase in the Young’s modulus can be observed (Table 5). The average UTS of CFF/Onyx_N2+FO150 is 9% lower than that of CFF/Onyx_N_2_, while the average UTS of both GFF/Onyx_N2+FO150 and KFF/Onyx_N2+FO150 increases by approximately 7%. Remarkably, GFF/Onyx_N2+FO150 exhibits an average UTS of 399 MPa, exceeding the highest recorded UTS of CFF/Onyx in this study (i.e., 376 MPa) by 6.1%.

#### 3.1.4. Deformation Mechanisms of CFRP Specimens

**Figure 5:**
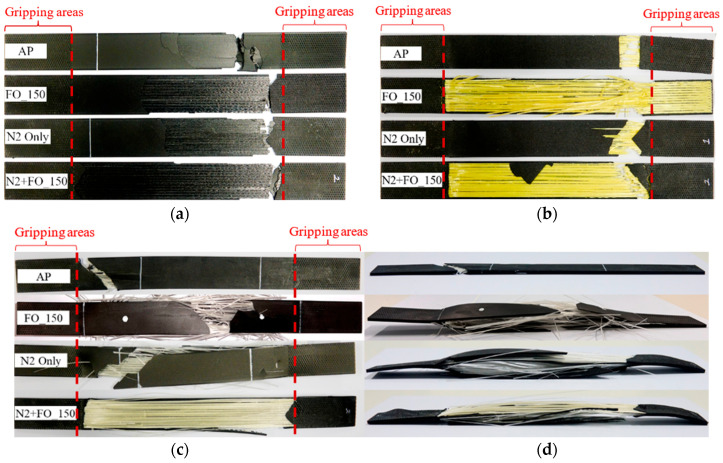
Top view of fractured specimens: (**a**) CFF/Onyx specimens; (**b**) KFF/Onyx specimens; (**c**) GFF/Onyx specimens; (**d**) isometric view of fractured GFF/Onyx specimens. All the tensile composite specimens originally measured 250 × 25 × 2.5 mm^3^.

Top and isometric views of the fractured tensile specimens are displayed in Figure 5, and typical failure modes of CFF/Onyx, GFF/Onyx, and KFF/Onyx specimens are summarised in Table 6 according to Tensile Test Failure Codes in ASTM D3039 [38].

According to the top view of CFF/Onyx specimens (Figure 5a), there is almost no fibre pull-out, and specimens in all groups fail suddenly at the ultimate tensile load. The primary failure mechanisms are long-splitting and Onyx-fibre layer delamination for all groups. Conversely, delamination occurs in both N_2_-purged and post-annealed CFF/Onyx specimens. The CFF/Onyx_N_2_+FO150 specimens exhibit the most severe delamination between the Onyx and CFF layers. As for KFF/Onyx specimens (Figure 5b), the AP specimens fail with a transverse fracture surface, whilst all other KFF/Onyx specimens show angled fracture surfaces. Fibre pull-out happens in all groups. Moreover, when the specimens are post-annealed, delamination occurs between the Onyx and KFF layers. In the top view of GFF/Onyx specimens (Figure 5c), the AP specimens exhibit an angled fracture mode with fibre pull-out, while the N_2_-purged or post-annealed specimens display explosive failure with Onyx-fibre layer delamination and fibre layer splitting. For the specimens that failed in explosive mode, a crack is first generated at the middle of the gauge length along the longitudinal direction during the tests, and then the delamination between the Onyx and fibre layers occurs across the entire gauge area (Figure 5d).

#### 3.1.5. Ashby Diagram of the Tensile Properties of CFRPs

**Figure 6:**
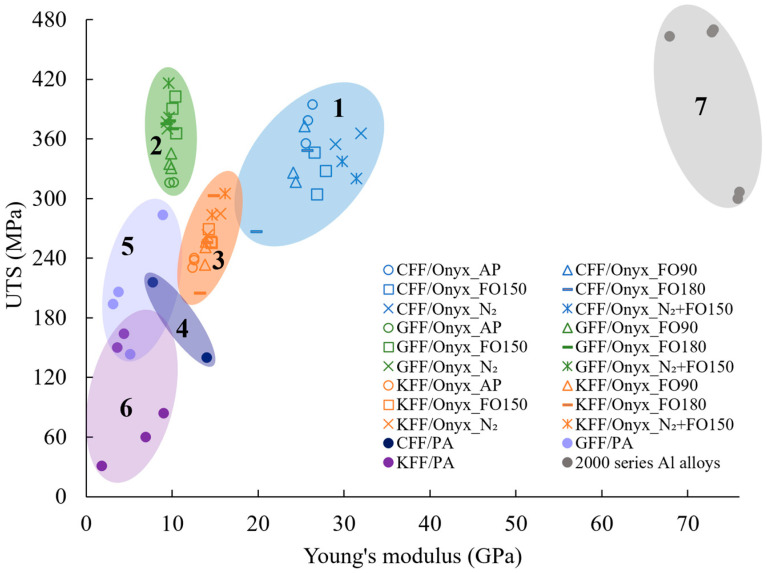
Ashby diagram showing the UTS versus Young’s modulus of CFF/Onyx (Region 1), GFF/Onyx (Region 2), and KFF/Onyx (Region 3) (from this study); CFF/PA (Region 4) [12,40], GFF/PA (Region 5) [12,41], and KFF/PA (Region 6) [12,42]; and 2000 series Al alloys (Region 7) [43,44,45]. Please note that solid symbols represent the data from literature.

The Ashby diagram presented in Figure 6 compares the Young’s modulus and UTS of the materials investigated in this study with those reported by other researchers [12,40,41,42,43,44,45]. In general, the CFRPs in the current study, falling in Regions 1–3, demonstrate superior tensile performance over MEX-printed continuous fibre-reinforced PA composites (Regions 4–6, with the data from literature, with PA/Nylon being the matrix of Onyx feedstock filaments), with higher values in both Young’s modulus and UTS. Furthermore, after N_2_-purging and post-annealing, the UTS of GFF/Onyx composites (Region 2) could be as high as 420 MPa, which falls into the upper range of the UTS values of 2000 series aluminium (Al) alloys commonly used in aerospace manufacturing [46] (Region 7 with the data from literature). Among the CFRPs in this study, the CFF/Onyx CFRPs (Region 1) stand out for their excellent Young’s modulus and UTS. Nevertheless, the Young’s modulus of the CRFCs remains low compared with aluminium alloys.

### 3.2. SEM Analysis

Microstructures were observed at both (1) the fracture surfaces of Onyx_AP and Onyx_FO150, and CFF/Onyx_AP and CFF/Onyx_FO150 specimens (Section 3.2.1), and (2) the individual carbon fibres in a CFF/Onyx_FO150 specimen, as well as the fracture surfaces of CFF/Onyx_FO180 and KFF/Onyx_FO180 specimens (Section 3.2.2).

#### 3.2.1. Fracture Behaviours of Neat Onyx and CFF/Onyx Under AP and FO150 Conditions

**Figure 7:**
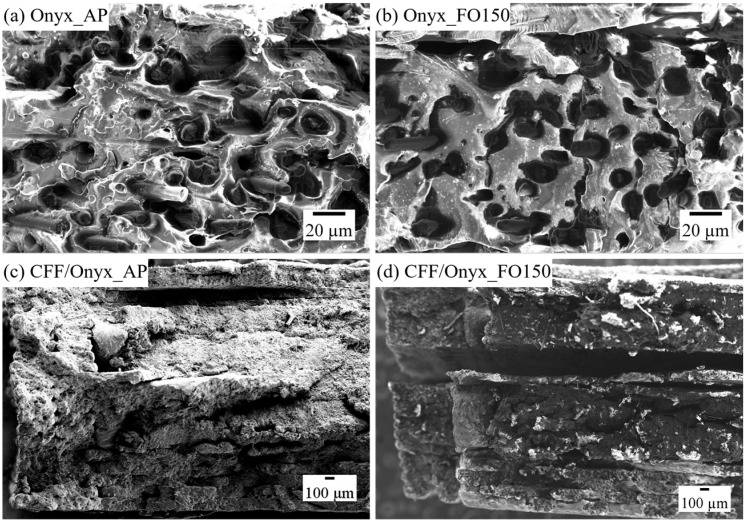
SEM images of partial fracture surfaces of tensile-tested specimens: (**a**) Onyx_AP; (**b**) Onyx_FO150; (**c**) CFF/Onyx_AP; (**d**) CFF/Onyx_FO150.

Figure 7 presents SEM images of partial fracture surfaces for neat Onyx and CFF/Onyx specimens under AP and FO150 conditions. In Figure 7a, the Onyx_AP specimen shows a rough fracture surface with a “wet” and porous morphology, suggesting good wettability and fusion of Onyx layers. After annealing at 150 °C for two hours (Figure 7b), the fracture surface becomes denser and smoother, indicating reduced ductility and a more brittle fracture behaviour. Figure 7c shows that the CFF/Onyx_AP specimen exhibits a rough and viscous fracture surface, with better fusion between the CFF and Onyx layers, reflecting effective load transfer and fibre filament. In contrast, the CFF/Onyx_FO150 specimen in Figure 7d displays a clean and brittle fracture surface with excessive delamination between CFF and Onyx layers. These changes suggest interfacial degradation between CFF and Onyx layers caused by thermal exposure.

Overall, the morphological differences between CFF/Onyx_AP and CFF/Onyx_FO150 confirm that post-annealing weakens the CFF-Onyx bonding and promotes premature failure.

#### 3.2.2. Post-Annealing Effects on Carbon Fibres, CFF/Onyx_FO180, and KFF/Onyx_FO180

**Figure 8:**
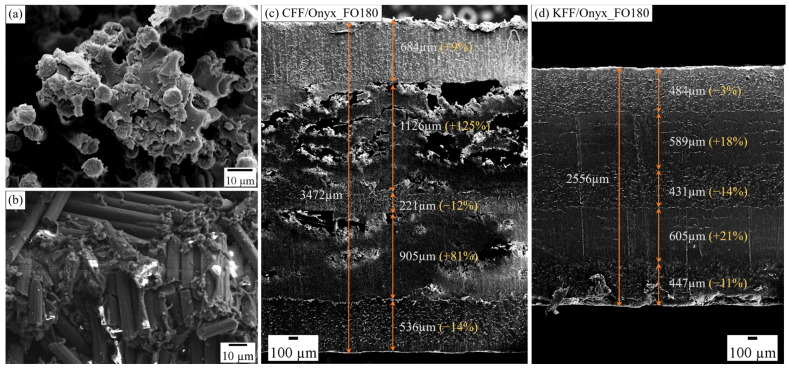
(**a**,**b**) SEM images of the fracture surface of a CFF/Onyx_FO150 specimen (**a**) brittle failure of individual carbon fibres; (**b**) carbon fibres broken into short segments; and (**c**,**d**) SEM images of partial cross-sections of fractured specimens, showing layer-wise morphology along the build direction: (**c**) CFF/Onyx_FO180; (**d**) KFF/Onyx_FO180 specimens. The thickness and the corresponding relative change are also indicated in the brackets for each layer.

The brittle failure of the CFF/Onyx specimen is associated with the sudden failure of individual carbon fibres within the CFF bundle, which can be observed in Figure 8a. Figure 8b shows that individual carbon fibres in a CFF/Onyx_FO150 specimen broke into short segments due to the embrittlement after annealing [47]. This explains the drop in tensile properties observed for CFF/Onyx specimens after post-printing annealing. Furthermore, based on the visual inspection and thickness measurements of the tensile specimens, significant thermal expansion was observed in the CFF/Onyx_FO180 specimens, whereas all specimens under FO90 and FO150 conditions exhibited minimal dimensional changes of less than 3%. Notably, one CFF/Onyx_FO180 specimen showed a substantial thickness increase of 37.7%. For comparison, Figure 8c,d show the SEM images of fractured edges, which expose the internal fibre and Onyx layers along the build direction, representing partial cross-sections of CFF/Onyx_FO180 and KFF/Onyx_FO180.

The extensive structural damage associated with thermal expansion and delamination in the CFF/Onyx specimens is mainly caused by the difference in the coefficients of thermal expansion (CTE) of CFF and Onyx. Representative CTE values in the in-plane (longitudinal and transverse) and through-thickness directions obtained from various papers and websites are summarised in Table 7. Previous studies have shown that fibre composites can exhibit a highly anisotropic CTE. The mismatch in thermal expansion, both between the fibres and the impregnating resin, and between the fibre-reinforced and matrix layers, can generate significant residual stresses during annealing, leading to internal cracking and interfacial delamination, ultimately causing premature failure of the composite parts [48,49,50,51]. As a result, post-annealing at 180 °C induces extensive cracking and the formation of large voids throughout the cross-section of CFF/Onyx, particularly within CFF layers, as illustrated in Figure 8c.

The longitudinal deformation difference between the fibre layers and the Onyx layer is expected to be comparable for both carbon fibre and Kevlar fibre layers because the values of the longitudinal CTE of carbon fibres and Kevlar fibres are similar. However, the difference in transverse CTE between carbon fibres and Onyx is considerably larger than that between Kevlar fibres and Onyx, which results in the distortion of CFF/Onyx specimens during heat treatment and leads to delamination when the mismatch between Onyx and CFF layers prevails on the interlayer bonding strength. According to the values in Table 7, the large through-thickness CTE of Onyx (approximately 248 × 10^−6^/°C) compared to carbon fibres (less than 10 × 10^−6^/°C) likely induces interlaminar residual stresses during annealing and cooling processes [52]. These stresses promote delamination and cracking, particularly in the CFF/Onyx_FO180 specimen. Moreover, the high thermal conductivity of carbon fibres (exceeding 84.4 W m^−1^ K^−1^ for individual carbon fibres [53]), as opposed to the relatively low thermal conductivity of Onyx (estimated between 0.3 and 0.9 W m^−1^ K^−1^ depending on the print direction [54]), limits heat transfer to underlying layers during annealing, distributing most of the thermal energy within the CFF layers and impeding effective interlayer bonding. This mechanism contributes to poor adhesion and delamination between CFF and Onyx layers [27].

Interestingly, the difference in CTE between Onyx and Kevlar fibres is also remarkable (Table 7), and yet the KFF/Onyx specimens did not experience any appreciable cracking or delamination. One important reason for this lies in the different mechanical properties of carbon and Kevlar fibres. Taking into consideration that the composite specimens under exam behave as elastic multilayer systems, and that Onyx layers are identical in CFF/Onyx and KFF/Onyx specimens, the thermal stresses in the fibre-reinforced layers are roughly proportional to their Young’s modulus [55], where the Young’s modulus of CFF is more than twice as much the Young’s modulus of KFF (Table 1).

Ultimately, it is worth noting that, although the Onyx matrix is nominally the same across all composite types, the resin used to impregnate the fibres may vary. The continuous carbon, glass, and KFF produced by Markforged^®^ are reported to be impregnated with distinct polyamide-based or proprietary thermoplastics, which may differ in their CTEs and interfacial adhesion characteristics compared to the Onyx matrix [56,57,58]. Additionally, the precise composition of the impregnating polymer remains proprietary and undisclosed [12]. Such resin mismatches could contribute to differential thermal behaviour and exacerbate internal stress accumulation during post-annealing, thereby promoting interfacial degradation in CFF/Onyx specimens.

**Table 7:** Summary of CTE values in three directions for printed Onyx parts, individual carbon fibres, glass fibres, and Kevlar fibres.

Materials	$\mathrm{CTE}\;\mathrm{Range}\;[\mathrm{Min},\;\mathrm{Max}]\;(10^{-6}/{}^{\circ}C$)		
	Longitudinal	Transverse	Through-Thickness
Printed Onyx parts [59]	[36, 46]	95	248
Carbon fibres [52,60,61,62]	[−1, 1]	[2.4, 36]	[5.9, 7.7]
Glass fibres [63]	[5, 12]	-	-
Kevlar fibres [64,65,66,67]	[−5.7, −2.7]	[66.3, 75]	-

### 3.3. DSC Analysis

**Figure 9:**
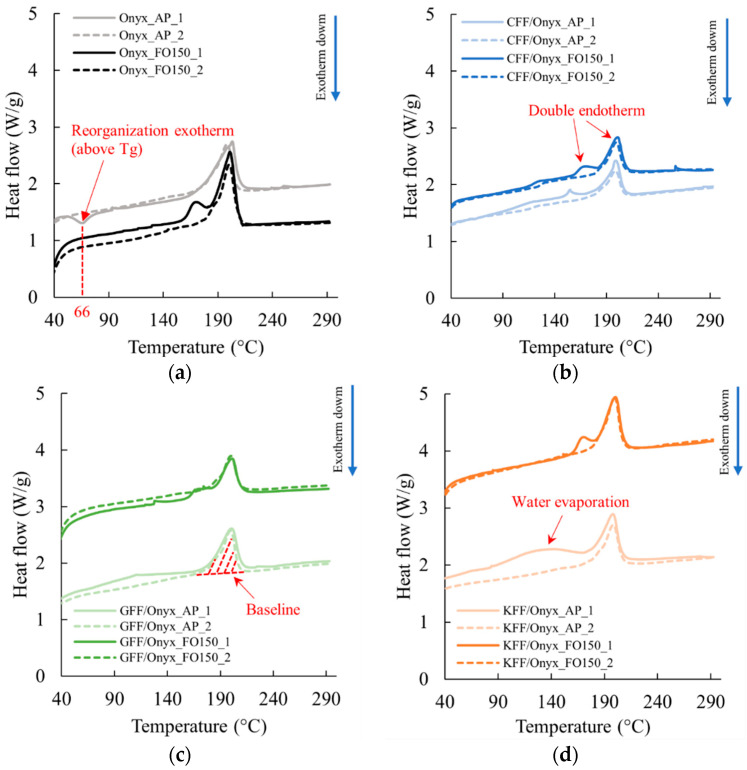
DSC curves of (**a**) Onyx; (**b**) CFF/Onyx; (**c**) GFF/Onyx; (**d**) KFF/Onyx specimens under both AP and FO150 conditions.

The DSC curves of Onyx, CFF/Onyx, GFF/Onyx, and KFF/Onyx specimens under both AP and FO150 conditions are presented in Figure 9, where the labels “1” and “2” refer to the first and second heating runs, respectively. For the Onyx_AP specimen, whose thermogram is shown in Figure 9a, there is a small exothermic peak at approximately 66 °C, which is slightly above the glass transition temperature of PA6 (T_g_ = 64.5 °C [36]), indicating a structural relaxation [68]. Plausibly, this peak is not observed in the Onyx_FO150 specimen because molecular chains have already experienced a structural rearrangement during the annealing process, which results in a more stable and ordered molecular structure. Moreover, a double endotherm is observed during the first heating run for all the specimens under FO150 conditions (Figure 9b). In principle, the occurrence of this double peak may be attributed to a few factors, including melting of two different phases or melting of a single phase with different degrees of structural order [69,70]. This aspect will be discussed in more detail on the grounds of the XRD results presented in Section 3.4.

By comparing the curves of the AP specimens to the corresponding FO150 specimens, in the first heating run, the curves of the AP specimens, especially for the GFF/Onyx and KFF/Onyx specimens, show broad, relatively weak endothermic peaks starting from approximately 100 °C. These peaks are caused by the evaporation of the absorbed water inside the specimens [71]. Notably, these endothermic peaks are completely absent in the first run heating curves of the FO150 specimens, which demonstrates the potential benefit of post-annealing in reducing the moisture content of the printed specimens.

Relevant thermal properties of the printed specimens are derived from DSC curves and summarized in Table 8. The melting point corresponds to the temperature at peak heat flow, while the “melting peak” (enthalpy of fusion) is determined by integrating the peak area above the baseline (Figure 9c). According to the results for the first heating run in Table 8, the changes in melting point caused by post-annealing are negligible, whilst the melting peak of FO150 specimens after post-annealing is always higher than that of the corresponding AP specimen, which is a cue of a higher degree of crystallinity [27]. This enhanced crystallinity suggests better polymer chain alignment, improving stress transfer and load-bearing capacity [72].

### 3.4. XRD Analysis

The presence of the double endotherm peak in the DSC curves of all FO150 specimens (Figure 9) indicated a need to clarify the phase composition of the PA matrix. Based on the double peaks observed at around 170 °C and 200 °C in the thermograms (Figure 9a), three temperatures of 150 °C, 180 °C, and 220 °C were selected to anneal neat Onyx specimens for two hours. The specimens, labelled as Onyx_FO150, Onyx_FO180, and Onyx_FO220, were submitted for XRD analysis. In addition, a neat Onyx_AP specimen and a GFF/Onyx_AP specimen were also analysed by XRD.

**Figure 10:**
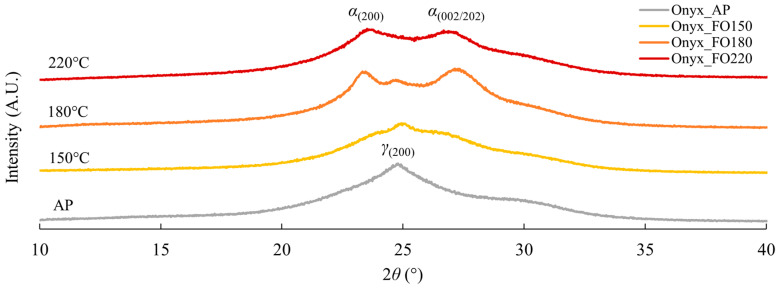
Diffractograms of neat Onyx specimens treated by various annealing temperatures.

The diffractograms (in the 10–40° 2*θ* range) are shown in Figure 10. The *γ* polymorph is the dominant phase in the Onyx_AP specimens. When subjected to post-annealing at 150 °C, the crystallinity increases, and the *γ* phase is still dominant, with a minor fraction of *α* phase present, as evidenced by weak peaks at each side of the *γ*_(200)_ peak. At 180 °C, the *γ* phase almost vanishes, whilst the *α*_(200)_ and *α*_(002/202)_ peaks become dominant. When the annealing temperature increases to 220 °C, the *γ* phase disappears with only the *α*_(200)_ and *α*_(002/202)_ peaks being visible in the diffractograms. This matches very well the thermal behaviours of Onyx and PA6 described by Handwerker et al. [27] and Millot et al. [73], respectively. In the research conducted by Millot et al. [73], the thermogram of a PA6 specimen annealed at 200 °C presented a double endotherm similar to Figure 9. Through wide-angle X-ray scattering (WAXS), Millot et al. [73] concluded that the double endotherm was caused by the melting of two different polymorphs, namely *α* crystallites and *γ* crystallites.

Table 9 lists the peak positions and the corresponding lattice spacings along with the crystallinity obtained using DIFFRAC.EVA software (version 6.1). Analogous data were also calculated for Onyx_AP and GFF/Onyx_AP specimens. An increase in crystallinity is observed for Onyx_FO150 specimens, achieving the maximum crystallinity among all tested temperatures. This enhancement in crystallinity can be attributed to the increased chain mobility due to relaxation of the amorphous phase in PA6 [27] along with interdiffusion between adjacent Onyx layers during post-annealing. The resulting chain entanglement across the interface strengthens interfacial bonding, a phenomenon known as autohesion, as reported by Awaja [74]. In addition, XRD results indicate the presence of both α and γ crystalline phases of PA6. These polymorphs exhibit distinct mechanical behaviours: the α-phase is associated with a more ordered crystalline structure, leading to improved stiffness and higher Young’s modulus, whereas the γ-phase, with its more loosely packed structure and greater interchain distance, contributes to increased ductility [75,76]. Therefore, the increased Young’s modulus and UTS of Onyx_FO150 specimens as discussed in Section 3.1.2 can be attributed to increased crystallinity and the favourable transformation of the crystalline phases. Subsequently, the crystallinity progressively decreases, with Onyx_FO180 exhibiting lower crystallinity and Onyx_FO220 showing the lowest. The calculated *d* values in Table 9 are consistent with the data reported in the literature for the *α* and *γ* polymorphs of PA6 [77,78], so the PA in the Onyx filaments can be reasonably identified as PA6. In particular, according to previous studies [27], the peaks at 2*θ* values of approximately 23.6° and 26.8° can be attributed to the *α* polymorph of PA6, whilst the *γ* polymorph exhibits a peak at around 24.8°.

Furthermore, the XRD analysis reveals remarkable similarities in phase composition between the GFF/Onyx_AP and Onyx_AP, where the diffractograms are presented in Appendix C. It is therefore very likely that the PA6 in Onyx plays a predominant role in the phase transitioning and crystalline structures of CFRPs as well. Moreover, the double endotherm in the first heating run of the DSC curves of all FO150 specimens in Figure 9 is most likely attributable to the melting processes of different PA6 phases during the first heating run. Finally, the absence of double peaks in the second heating curves is caused by the complete melting of all the phases after the first heating run ramping up to 300 °C, whereafter the material recrystallises to the *γ*_(200)_ phase.

## 4. Conclusions

This research examined the effects of N_2_ purging and post-annealing on the tensile properties of three types of CFRPs produced by MEX additive manufacturing, namely: carbon fibre-reinforced Onyx (CFF/Onyx), glass fibre-reinforced Onyx (GFF/Onyx), and Kevlar fibre-reinforced Onyx (KFF/Onyx). The key findings from this research are as follows:

- N_2_-purging, post-annealing, and their combination (N_2_+FO150) improved the Young’s modulus and UTS of KFF/Onyx specimens;
- For GFF/Onyx, N_2_-purging, post-annealing, and their combination (N_2_+FO150) had a very minor effect (no more than 6%) on the Young’s modulus, while positive effects were observed for the UTS. Remarkably, the UTS of GFF/Onyx reached an average value of 399 MPa following the application of the combined treatment, surpassing the highest average UTS of (as printed) CFF/Onyx by over 6%. Moreover, the UTS of GFF/Onyx was comparable to that of 2000 series aluminium alloys. This finding highlighted the efficacy of the combined treatment in significantly improving the mechanical performance of GFF/Onyx, offering valuable implications for additive manufacturing. Specifically, GFF/Onyx, after combined treatment, presented potential for substituting traditional metal materials, contributing to weight reduction in relevant industrial applications such as aerospace manufacturing;
- For CFF/Onyx specimens, N_2_-purging had a substantial positive effect on the Young’s modulus. On the other hand, post-annealing at 90 °C and 150 °C had negligible effects on the Young’s modulus, while post-annealing at 180 °C reduced the Young’s modulus. The combined treatment (N_2_+FO150) resulted in similar effects on the Young’s modulus. For UTS of CFF/Onyx, all treatments, including N_2_-purging, post-annealing, and their combination, reduced the UTS of CFF/Onyx. Scanning electron microscopy (SEM) analysis revealed large interlayer gaps and fibre detachment after annealing at 150 °C. At 180 °C, post-annealing induced thermal expansion, embrittlement of carbon fibres, and crack propagation, all contributing to decreased UTS in CFF/Onyx specimens;
- The different effects of N_2_-purging, post-annealing, and their combination (N_2_+FO150) on the Young’s modulus and UTS of CFF/Onyx, GFF/Onyx, and KFF/Onyx suggested that the response of CFRPs to N_2_-purging and post-annealing was material-dependent, emphasising the need for a targeted approach in optimising the mechanical properties of additively manufactured composite materials;
- DSC analysis of as-printed (AP) and post-annealed (FO150) Onyx, CFF/Onyx, GFF/Onyx, and KFF/Onyx specimens revealed structural relaxation, increased crystallinity, double endothermic peaks, and reduced moisture content after annealing at 150 °C. These observations indicate enhanced molecular stability and load-bearing capability. XRD analysis confirmed the polyamide component as PA6 and attributed the double endothermic peaks observed in DSC curves to the melting of distinct PA6 crystalline phases. Both DSC and XRD analyses consistently demonstrated increased crystallinity in the specimens annealed at 150 °C.

Future research will investigate the impact of N_2_-purging and post-annealing on diverse mechanical properties of CFRPs, including compression and three-point bending. Such multifaceted exploration will contribute to a comprehensive understanding of the thermal and mechanical behaviour of additively manufactured CFRPs, thereby improving the fabrication processes and enabling wider industrial applications.

## Figures and Tables

**Table 1:** Characteristics of Onyx and fibre filaments from Markforged datasheets [35].

Material	Onyx	CFF	GFF	KFF
Tensile strength (MPa)	40	800	590	610
Young’s modulus (GPa)	2.4	60	21	27
Failure strain (mm/mm) ^1^	0.250	0.015	0.038	0.027
Heat deflection temperature (°C)	145	105	105	105
Density (g/cm^3^)	1.2	1.4	1.5	1.2

^1^ All failure strain values reported in this paper are calculated based on engineering strain and are expressed in unitless form (mm/mm).

**Table 2:** Detailed configuration of CFF/Onyx, GFF/Onyx, and KFF/Onyx specimens.

Specimen	Onyx Layers	Fibre-Reinforced Layers	Layer Thickness ^1^ (mm)	Onyx Volume ^2^ (cm^3^)	Fibre Filament Volume ^2^ (cm^3^)	Fibre Filament Vf
CFF/Onyx	1–5, 10–11, 16–20	6–9, 12–15	0.125	10.30	5.24	33.72%
GFF/Onyx	1–5, 11–15, 21–25	6–10, 16–20	0.1	10.32	5.35	34.14%
KFF/Onyx	1–5, 11–15, 21–25	6–10, 16–20	0.1	10.32	5.45	34.56%

^1^ Values are for both fibre-reinforced and Onyx layers. ^2^ Values were calculated by the slicing software, Eiger.

**Table 3:** Tensile properties of as-received and annealed feedstock fibre filaments.

Fibre Filament	Average Diameter (mm)	Young’s Modulus (GPa)	UTS (MPa)	Failure Strain (mm/mm)
CFF_as-received_	0.363	48 ± 3	1494 ± 44	0.04 ± 0.00
CFF_annealed_	0.379	49 ± 3	978 ± 233	0.02 ± 0.01
GFF_as-received_	0.332	16 ± 1	516 ± 46	0.03 ± 0.00
GFF_annealed_	0.293	25 ± 2	1196 ± 134	0.05 ± 0.01
KFF_as-received_	0.315	27 ± 1	625 ± 72	0.02 ± 0.00
KFF_annealed_	0.305	35 ± 3	1070 ± 76	0.03 ± 0.00

**Table 4:** Tensile properties of neat Onyx specimens under various scenarios.

Specimen	Young’s Modulus (GPa)	UTS (MPa)	Failure Strain (mm/mm)
AP	0.94 ± 0.05	34 ± 1	0.26 ± 0.03
N_2_	1.34 ± 0.14	37 ± 1	0.20 ± 0.02
FO150	1.49 ± 0.00	50 ± 1	0.16 ± 0.01
N_2_+FO150	2.58 ± 0.01	49 ± 1	0.10 ± 0.01

**Table 5:** Summary of tensile properties for all types of CFRPs.

Material	Scenario	Young’s Modulus (GPa)	UTS (MPa)	Failure Strain (mm/mm)
CFF/Onyx	AP	25.89 ± 0.32	376.27 ± 15.98	0.015 ± 0.001
	N_2_	30.50 ± 1.47	360.27 ± 5.39	0.012 ± 0.000
	FO90	24.63 ± 0.56	338.59 ± 24.46	0.014 ± 0.001
	FO150	27.16 ± 0.56	325.54 ± 17.18	0.012 ± 0.001
	FO180	22.83 ± 2.96	307.22 ± 40.97	0.017 ± 0.003
	N2+FO150	30.61 ± 0.82	328.89 ± 8.63	0.011 ± 0.000
GFF/Onyx	AP	9.93 ± 0.24	316.20 ± 0.02	0.034 ± 0.000
	N_2_	9.38 ± 0.05	373.49 ± 3.33	0.042 ± 0.000
	FO90	9.84 ± 0.09	337.01 ± 6.33	0.034 ± 0.001
	FO150	10.35 ± 0.18	385.75 ± 15.43	0.038 ± 0.002
	FO180	9.78 ± 0.29	374.24 ± 3.27	0.039 ± 0.001
	N2+FO150	9.63 ± 0.03	399.00 ± 17.19	0.044 ± 0.002
KFF/Onyx	AP	12.50 ± 0.08	236.33 ± 4.09	0.020 ± 0.000
	N_2_	14.87 ± 0.76	274.07 ± 10.76	0.019 ± 0.000
	FO90	13.88 ± 0.04	247.06 ± 9.94	0.018 ± 0.000
	FO150	14.50 ± 0.15	260.09 ± 6.34	0.018 ± 0.000
	FO180	14.09 ± 0.65	254.00 ± 40.09	0.018 ± 0.002
	N2+FO150	15.43 ± 0.75	294.33 ± 10.76	0.020 ± 0.000

**Table 6:** Summary of failure modes for specimens across three types of CFRPs under AP, FO150, N_2_, and N_2_+FO150 conditions.

Material	Scenario	Failure Mode	Description
CFF/Onyx	AP	LGT	L-lateral failure, G-gauge area, T-top location
	FO150	M(xyz)GT	M(xyz)-multi-modes, G-gauge area, T-top location
	N_2_	M(xyz)GT	M(xyz)-multi-modes, G-gauge area, T-top location
	N_2_+FO150	M(xyz)GT	M(xyz)-multi-modes, G-gauge area, T-top location
GFF/Onyx	AP	AGB	A-angled failure, G-gauge area, B-bottom location
	FO150	XGM	X-explosive failure, G-gauge area, M-middle location
	N_2_	XGV	X-explosive failure, G-gauge area, V-various locations
	N_2_+FO150	XGV	X-explosive failure, G-gauge area, V-various locations
KFF/Onyx	AP	LGT	L-lateral failure, G-gauge area, T-top location
	FO150	AGB	A-angled failure, G-gauge area, B-bottom location
	N_2_	XGV	X-explosive failure, G-gauge area, V-various locations
	N_2_+FO150	M(xyz)GB	M(xyz)-multi-modes, G-gauge area, B-bottom location

**Table 8:** DSC results for Onyx, CFF/Onyx, GFF/Onyx, and KFF/Onyx specimens.

Material	Scenario	Melting Point (°C)		Melting Peak (J/g)	
		1st Heating Run	2nd Heating Run	1st Heating Run	2nd Heating Run
Onyx	AP	203	198	18.6	14.3
	FO150	201	200	26.8	18.5
CFF/Onyx	AP	199	198	8.0	7.1
	FO150	201	199	13.1	8.5
GFF/Onyx	AP	200	199	13.2	11.8
	FO150	201	200	14.1	10.2
KFF/Onyx	AP	198	198	12.0	12.0
	FO150	200	200	20.8	15.0

**Table 9:** XRD results of Onyx specimens being post-annealed at various temperatures and the GFF/Onyx_AP specimen.

Specimen	2*θ* at Peak (°)	*d* Spacing (nm)	$X_{c}$ ^1^ (%)
Onyx_AP	24.77	0.417	22.0
Onyx_FO150 (1st peak)	23.76	0.435	24.4
Onyx_FO150 (2nd peak)	24.98	0.414	
Onyx_FO150 (3rd peak)	26.34	0.393	
Onyx_FO180 (1st peak)	23.44	0.441	21.4
Onyx_FO180 (2nd peak)	24.63	0.420	
Onyx_FO180 (3rd peak)	27.17	0.381	
Onyx_FO220 (1st peak)	23.67	0.436	16.0
Onyx_FO220 (2nd peak)	26.81	0.386	
GFF/Onyx_AP	24.97	0.414	20.8

^1^ $X_{c}$ refers to the degree of crystallinity of specimens calculated by DIFFRAC.EVA software.

**Figure A1:**
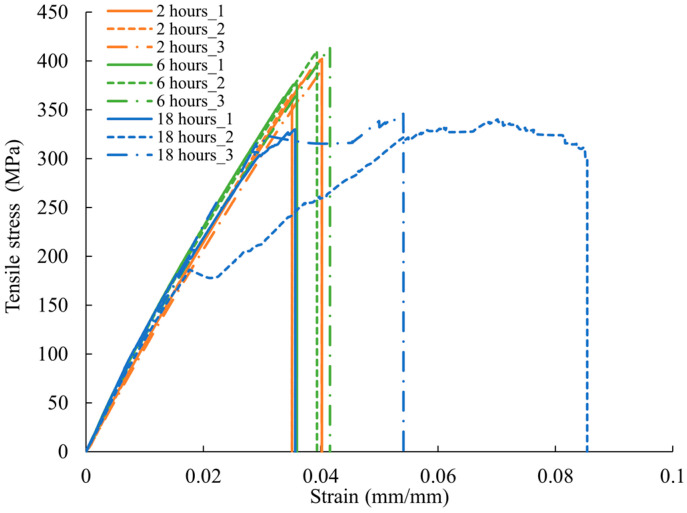
Stress-strain curves of GFF/Onyx specimens subjected to post-annealing for two, six, and 18 h.

**Figure A2:**
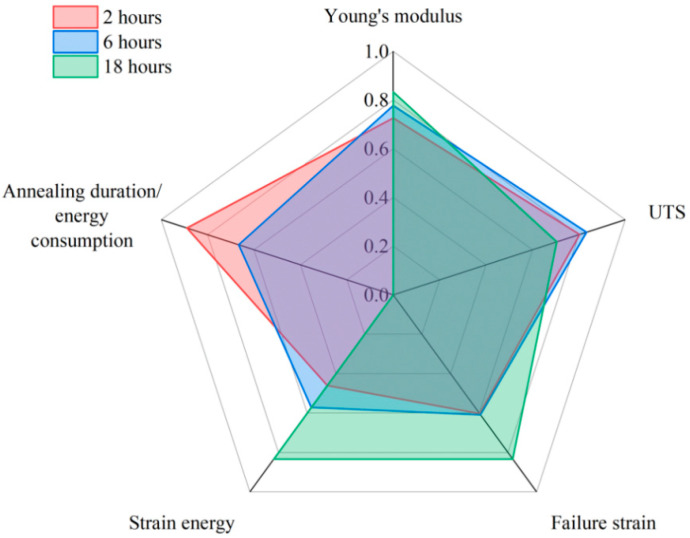
Spider diagram of the normalised indicators of the GFF/Onyx specimens subjected to post-annealing at 150 °C for two, six, and 18 h.

**Figure A3:**
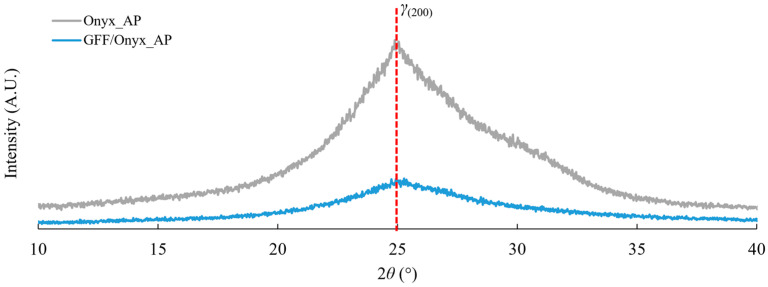
Diffractograms of Onyx_AP and GFF/Onyx_AP specimens.

## Data Availability

The original contributions presented in this study are included in the article. Further inquiries can be directed to the corresponding author.

## Appendix A. Chamber Temperature (CT) and Relative Humidity (RH) Recordings

**Table A1:** CT and RH during carbon fibre-reinforced Onyx (CFF/Onyx) specimen printing.

Specimen	AP	N_2_	FO90	FO150	FO180	N_2_+FO150
CT (°C)	35.72	36.87	35.21	36.98	35.21	36.10
RH (%)	27.08	3.10	32.46	28.67	32.46	7.52

Note: AP: as-printed specimens; N_2_: specimens printed with N_2_-purging; FO90, FO150 and FO180: specimens annealed at 90 °C, 150 °C and 180 °C for two hours; N_2_+FO150: specimens printed with N_2_-purging and annealed at 150 °C for two hours after printing.

**Table A2:** CT and RH during glass fibre-reinforced Onyx (GFF/Onyx) specimen printing.

Specimen	AP	N_2_	FO90	FO150	FO180	N_2_+FO150
CT (°C)	34.62	34.89	32.31	31.79	35.13	35.68
RH (%)	29.68	4.49	28.41	37.20	29.66	7.19

**Table A3:** CT and RH during Kevlar fibre-reinforced Onyx (KFF/Onyx) specimen printing.

Specimen	AP	N_2_	FO90	FO150	FO180	N_2_+FO150
CT (°C)	36.18	36.40	37.17	36.18	34.28	34.66
RH (%)	24.05	11.04	23.21	24.05	24.04	5.24

## Appendix B. Evaluation of Post-Annealing Duration

To investigate the effect of annealing duration, GFF/Onyx specimens were post-annealed at 150 °C for two, six, and 18 h, respectively. Five indicators were selected to characterise the properties of post-annealed specimens, namely Young’s modulus, UTS, failure strain, strain energy, and annealing duration/energy consumption. The stress-strain curves of the post-annealed GFF/Onyx specimens are shown in Figure A1. The failure strain is the maximum strain that each specimen reached at the point of fracture or failure. The strain energy was calculated by integrating the area under the stress-strain curve up to the failure strain for each specimen. Additionally, energy consumption was determined by multiplying the annealing duration by the power of the fan oven dryer, which was assumed to be 0.8 kW according to the oven’s specifications.

Particularly, the unconventional shape of the “18 h_2” curve for the GFF/Onyx specimen is likely due to excessive deformation and premature failure resulting from material degradation after prolonged annealing. Compared to the specimens annealed for two and six hours, which exhibited typical linear-elastic behaviour followed by brittle failure, the 18-h specimen showed signs of matrix softening and potential fibre-matrix debonding. These effects likely impaired stress transfer efficiency and promoted unstable crack propagation, leading to the irregular, wavy stress-strain response observed. Similar degradation effects have been reported in conventionally manufactured fibre-reinforced composites. Salvi et al. [79] observed that prolonged annealing (ranging from 16 to 192 h) significantly reduces both flexural strength and strain at maximum load in short glass fibre-reinforced PA66, with the extent of degradation increasing with annealing duration. While short-term ageing may initially enhance flexural strength due to stress relaxation and annealing effects, prolonged thermal exposure results in deterioration driven by thermal oxidation. In addition, Liao et al. [80] also reported that prolonged thermal ageing up to 4000 h at 95 °C induces interfacial degradation in injection-moulded glass fibre-reinforced epoxy composites. While their study focused on long-term exposure at a lower temperature, the underlying degradation mechanisms were consistent with the interfacial damage and specimen deformation observed in the 18-h GFF/Onyx specimen. These findings suggest that long-term annealing effects on AM CFRPs require further investigation.

The indicators for the post-annealed GFF/Onyx specimens and the best and worst values for each indicator are provided in Table A4. For the Young’s modulus, the UTS, the failure strain and the strain energy, the best and worse values were determined, respectively, as 120% of the highest values among 2-h, 6-h and 18-h annealing, and zero; for the annealing duration and energy consumption, the best values are 0, while the worst values are the longest duration and highest consumption.

**Table A4:** Indicators for the GFF/Onyx specimens subjected to post-annealing at 150 °C for two, six, and 18 h.

Indicator	2-h Annealing	6-h Annealing	18-h Annealing	Best Value	Worst Value
Young’s modulus (GPa)	10.4	11.1	11.9	14.3	0
UTS (MPa)	386	400	339	480	0
Failure strain [-]	0.039	0.039	0.053	0.064	0
Strain energy (J)	41.84	51.95	75.81	90.97	0
Annealing duration (hours)	2	6	18	0	18
Energy consumption (kWh)	1.6	4.8	14.4	0	14.4

For clearer comparisons, a normalisation method, Equation (A1) [81], was used to normalise the indicators, and the normalised indicators were plotted in the spider diagram in Figure A2.

(A1) $$\mathrm{Normalised}\;\mathrm{index}\;=\frac{\;\mathrm{Test}\;\mathrm{value}\;-\;\mathrm{Worst}\;\mathrm{value}\;}{\;\mathrm{Best}\;\mathrm{value}\;-\;\mathrm{Worst}\;\mathrm{value}\;}$$

According to Table A5, assigning an equal weighting factor (20%) to each indicator, post-annealing at 150 °C for two hours resulted in a final weighted score of 0.7 out of 1, demonstrating that it is an efficient method for achieving good tensile performance while consuming reasonable amounts of time and energy.

**Table A5:** Normalised indices and weighted scores of each indicator for GFF/Onyx subjected to post-annealing for two, six, and 18 h.

Parameter	2-h Annealing	6-h Annealing	18-h Annealing
Young’s modulus	0.73	0.78	0.83
UTS	0.80	0.83	0.70
Failure strain	0.60	0.61	0.83
Strain energy	0.46	0.57	0.83
Annealing duration/energy consumption	0.89	0.67	0.00
Weighted score	0.70	0.69	0.64

## References

[1] Barış Vatandaş B., Uşun A., Yıldız N., Şimşek C., Necati Cora Ö., Aslan M., Gümrük R. (2023). Additive manufacturing of PEEK-based continuous fiber reinforced thermoplastic composites with high mechanical properties. Compos. Part A Appl. Sci. Manuf..

[2] Subramaniyan M., Karuppan S., Appusamy A., Pitchandi N. (2025). Sandwich printing of PLA and carbon fiber reinforced-PLA for enhancing tensile and impact strength of additive manufactured parts. J. Manuf. Process..

[3] Wohlers T.T., Campbell R.I., Diegel O., Huff R., Kowen J. (2023). Wohlers Report 2023: Analysis. Trends. Forecasts. 3D Printing and Additive Manufacturing State of the Industry.

[4] Bezzina C.M., Refalo P. (2023). Fused Filament Fabrication and Injection Moulding of Plastic Packaging: An Environmental and Financial Comparative Assessment. Machines.

[5] Liu X., Shan Z., Liu J., Liu F., Wu X., Zou A., Du W., Wu S., Jiang X. (2024). Mechanical and dielectric properties of continuous glass fiber reinforced poly-ether-ether-ketone composite components prepared by additive manufacturing. Addit. Manuf..

[6] Wang Z., Zhou M., Jiang J., Huang H., Chen B., Li Y., Zhai W. (2024). Fabrication of PEI/CF composite parts with multi-angle reinforced mechanical properties by a micro-extrusion foaming FDM process. Compos. Part A Appl. Sci. Manuf..

[7] Cheng H., Tang M., Zhang J., Wang H., Zhou J., Wang Q., Qian Z. (2024). Effects of rCF attributes and FDM-3D printing parameters on the mechanical properties of rCFRP. Compos. Part B Eng..

[8] Tian X., Liu T., Yang C., Wang Q., Li D. (2016). Interface and performance of 3D printed continuous carbon fiber reinforced PLA composites. Compos. Part A Appl. Sci. Manuf..

[9] Marsavina L., Valean C., Marghitas M., Linul E., Razavi N., Berto F. (2022). Effect of the manufacturing parameters on the tensile and fracture properties of FDM 3D-printed PLA specimens. Eng. Fract. Mech..

[10] Li J., Durandet Y., Huang X., Sun G., Ruan D. (2022). Additively manufactured fiber-reinforced composites: A review of mechanical behavior and opportunities. J. Mater. Sci. Technol..

[11] Giani N., Mazzocchetti L., Benelli T., Picchioni F., Giorgini L. (2022). Towards sustainability in 3D printing of thermoplastic composites: Evaluation of recycled carbon fibers as reinforcing agent for FDM filament production and 3D printing. Compos. Part A Appl. Sci. Manuf..

[12] Dickson A.N., Barry J.N., McDonnell K.A., Dowling D.P. (2017). Fabrication of continuous carbon, glass and Kevlar fibre reinforced polymer composites using additive manufacturing. Addit. Manuf..

[13] Nguyen-Van V., Peng C., Tran P., Wickramasinghe S., Do T., Ruan D. (2024). Mechanical and dynamic performance of 3D-printed continuous carbon fibre Onyx composites. Thin-Walled Struct..

[14] Zaldivar R.J., Mclouth T.D., Ferrelli G.L., Patel D.N., Hopkins A.R., Witkin D. (2018). Effect of initial filament moisture content on the microstructure and mechanical performance of ULTEM^®^ 9085 3D printed parts. Addit. Manuf..

[15] Zhang Y., Zhou J., Qin R., Shi S., Lu Y., Zhang X., Xu J., Chen B. (2025). Defect analysis and quality evaluation system for additive manufactured continuous fiber-reinforced polymer composites. J. Manuf. Process..

[16] Li X., Liu Y., Li S., Wang K., Correia J.P.M., Ahzi S. (2025). Printing path induced temperature history and interfacial properties of 3D printed continuous nature fiber reinforced polypropylene composites. J. Manuf. Process..

[17] Valvez S., Reis P.N.B., Ferreira J.A.M. (2023). Effect of annealing treatment on mechanical properties of 3D-Printed composites. J. Mater. Res. Technol..

[18] Wickramasinghe S., Do T., Tran P. (2020). FDM-Based 3D Printing of Polymer and Associated Composite: A Review on Mechanical Properties, Defects and Treatments. Polymers.

[19] Vatandaş B.B., Uşun A., Gümrük R. (2024). Mechanical performances of continuous carbon fiber reinforced PEEK (polyether ether ketone) composites printed in a vacuum environment. J. Manuf. Process..

[20] Shaik Y.P., Schuster J., Naidu N.K. (2023). High-Pressure FDM 3D Printing in Nitrogen [Inert Gas] and Improved Mechanical Performance of Printed Components. J. Compos. Sci..

[21] Zheng L., Zhang Q., Yu X., Luo X., Jiang H. (2023). Effect of annealing and heat-moisture pretreatment on the quality of 3D-printed wheat starch gels. Innov. Food Sci. Emerg..

[22] Zhang Y., Lu J., Wu J., Gao X. (2024). Exploring the impact of cellulose nanocrystals and annealing treatment on the interlayer bond strength of polylactic acid 3D printed composites. J. Appl. Polym. Sci..

[23] Yu W., Wang X., Yin X., Ferraris E., Zhang J. (2023). The effects of thermal annealing on the performance of material extrusion 3D printed polymer parts. Mater. Des..

[24] Liesenfeld J., Jablonski J.J., da Silva J.R.F., Buenos A.A., Scheuer C.J. (2024). Impact of annealing on the characteristics of 3D-printed graphene-reinforced PLA composite. J. Manuf. Process..

[25] Chueca de Bruijn A., Gómez-Gras G., Fernández-Ruano L., Farràs-Tasias L., Pérez M.A. (2023). Optimization of a combined thermal annealing and isostatic pressing process for mechanical and surface enhancement of Ultem FDM parts using Doehlert experimental designs. J. Manuf. Process..

[26] Ahmad W., Khan H.A., Salik S., Ali H.Q., Khushbash S., Qureshi Z.A. (2024). Extrusion-based additive manufacturing of CFRP/steel/CFRP multi-material structure: Process development and influence of heat treatment on the mechanical performance. J. Manuf. Process..

[27] Handwerker M., Wellnitz J., Marzbani H., Tetzlaff U. (2021). Annealing of chopped and continuous fibre reinforced polyamide 6 produced by fused filament fabrication. Compos. Part B Eng..

[28] Wang P., Zou B. (2022). Improvement of Heat Treatment Process on Mechanical Properties of FDM 3D-Printed Short- and Continuous-Fiber-Reinforced PEEK Composites. Coatings.

[29] Muna I.I., Mieloszyk M., Rimasauskiene R., Maqsood N., Rimasauskas M. (2022). Thermal Effects on Mechanical Strength of Additive Manufactured CFRP Composites at Stable and Cyclic Temperature. Polymers.

[30] Markforged (2023). Markforged Mark Two Printer. https://markforged.com/3d-printers/mark-two.

[31] Le Duigou A., Barbé A., Guillou E., Castro M. (2019). 3D printing of continuous flax fibre reinforced biocomposites for structural applications. Mater. Des..

[32] Terekhina S., Egorov S., Tarasova T., Skornyakov I., Guillaumat L., Hattali M.L. (2022). In-nozzle impregnation of continuous textile flax fiber/polyamide 6 composite during FFF process. Compos. Part A Appl. Sci. Manuf..

[33] Tian X., Todoroki A., Liu T., Wu L., Hou Z., Ueda M., Hirano Y., Matsuzaki R., Mizukami K., Iizuka K. (2022). 3D Printing of Continuous Fiber Reinforced Polymer Composites: Development, Application, and Prospective. Chin. J. Mech. Eng..

[34] Markforged (2023). Micro Carbon Fiber Filled Nylon that Forms the Foundation of Markforged Composite Parts. https://markforged.com/materials/plastics/onyx?__geom=%E2%9C%AA.

[35] Markforged Markforged Material Datasheet—Composites. https://www-objects.markforged.com/craft/materials/CompositesV5.2.pdf.

[36] Spina R. (2019). Performance Analysis of Colored PLA Products with a Fused Filament Fabrication Process. Polymers.

[37] (2022). Standard Test Method for Tensile Properties of Plastics, International, A. https://compass.astm.org/document/?contentCode=ASTM%7CD0638-22%7Cen-US.

[38] (2022). Standard Test Method for Tensile Properties of Polymer Matrix Composite Materials, International, A. https://compass.astm.org/document/?contentCode=ASTM%7CD3039_D3039M-08%7Cen-US&proxycl=https%3A%2F%2Fsecure.astm.org&fromLogin=true.

[39] Li J., Xu S., Durandet Y., Gao W., Huang X., Ruan D. (2024). Strain rate dependence of 3D printed continuous fiber reinforced composites. Compos. Part B Eng..

[40] Klift F.V.D., Koga Y., Todoroki A., Ueda M., Hirano Y., Matsuzaki R. (2016). 3D Printing of Continuous Carbon Fibre Reinforced Thermo-Plastic (CFRTP) Tensile Test Specimens. Open J. Compos. Mater..

[41] Díaz-Rodríguez J.G., Pertúz-Comas A.D., González-Estrada O.A. (2021). Mechanical properties for long fibre reinforced fused deposition manufactured composites. Compos. Part B Eng..

[42] Melenka G.W., Cheung B.K.O., Schofield J.S., Dawson M.R., Carey J.P. (2016). Evaluation and prediction of the tensile properties of continuous fiber-reinforced 3D printed structures. Compos. Struct..

[43] Owolabi G.M., Thom M., Ajide O.O., Kumar N., Odeshi A.G., Warner G. (2019). Tensile Properties and Fractography of Three AA 2000 Series Aluminum Alloys Used for Aerospace Applications. Trans. Indian Inst. Met..

[44] Sandoval J.H., Mohamed A.M.A., Valtierra S., Samuel F.H. (2014). Mechanical Performance of a Cast A354 Aluminium Alloy. Mater. Sci. Forum.

[45] Merayo D., Rodríguez-Prieto A., Camacho A.M. (2020). Prediction of the Bilinear Stress-Strain Curve of Aluminum Alloys Using Artificial Intelligence and Big Data. Metals.

[46] Jin J., Geng S., Shu L., Jiang P., Shao X., Han C., Ren L., Li Y., Yang L., Wang X. (2024). High-strength and crack-free welding of 2024 aluminium alloy via Zr-core-Al-shell wire. Nat. Commun..

[47] Bertran X., Labrugère C., Dourges M.A., Rebillat F. (2013). Oxidation Behavior of PAN-based Carbon Fibers and the Effect on Mechanical Properties. Oxid. Met..

[48] Hahn H.T. (1976). Residual Stresses in Polymer Matrix Composite Laminates. J. Compos. Mater..

[49] Hahn H.T., Pagano N.J. (1975). Curing Stresses in Composite Laminates. J. Compos. Mater..

[50] Fletcher A.J., Oakeshott J.L. (1994). Thermal residual microstress generation during the processing of unidirectional carbon fibre/epoxy resin composites: Regular fibre arrays. Composites.

[51] Villeneuve J.F., Naslain R., Fourmeaux R., Sevely J. (1993). Longitudinal/radial thermal expansion and poisson ratio of some ceramic fibres as measured by transmission electron microscopy. Compos. Sci. Technol..

[52] Ahmed A., Tavakol B., Das R., Joven R., Roozbehjavan P., Minaie B. Study of Thermal Expansion in Carbon Fiber Reinforced Polymer Composites. Proceedings of the SAMPE International Symposium Proceedings.

[53] Wang Z.L., Tang D.W., Zhang W.G. (2007). Simultaneous measurements of the thermal conductivity, thermal capacity and thermal diffusivity of an individual carbon fibre. J. Phys. D Appl. Phys..

[54] Mulholland T., Felber R., Rudolph N. Design and Additive Manufacturing of a Composite Crossflow Heat Exchanger. Proceedings of the 28th Annual International Solid Freeform Fabrication Symposium—An Additive Manufacturing Conference.

[55] Hsueh C.H. (2002). Thermal stresses in elastic multilayer systems. Thin Solid Film..

[56] Lupone F., Padovano E., Venezia C., Badini C. (2022). Experimental Characterization and Modeling of 3D Printed Continuous Carbon Fibers Composites with Different Fiber Orientation Produced by FFF Process. Polymers.

[57] Ju J., Yang N., Yu L., Zhang Z., Jiang H., Wu W., Ma G. (2025). Experiment and Simulation Study on the Crashworthiness of Markforged 3D-Printed Carbon/Kevlar Hybrid Continuous Fiber Composite Honeycomb Structures. Materials.

[58] Saidane E.H., Arnold G., Louis P., Pac M.-J. (2022). 3D printed continuous glass fibre-reinforced polyamide composites: Fabrication and mechanical characterisation. J. Reinf. Plast. Compos..

[59] Faust J.L., Kelly P.G., Jones B.D., Roy-Mayhew J.D. (2021). Effects of Coefficient of Thermal Expansion and Moisture Absorption on the Dimensional Accuracy of Carbon-Reinforced 3D Printed Parts. Polymers.

[60] Chung D.D.L., Chung D.D.L. (2017). 7-Carbon-Matrix Composites. Carbon Composites.

[61] Christine (2023). Carbon Fiber Characteristics. https://www.christinedemerchant.com/carboncharacteristics.html.

[62] Wong T.G. (2023). Low Thermal Expansion. https://www.ngfworld.com/en/fiber/low_thermal_expansion.html.

[63] Chu X., Wu Z., Huang R., Zhou Y., Li L. (2010). Mechanical and thermal expansion properties of glass fibers reinforced PEEK composites at cryogenic temperatures. Cryogenics.

[64] Rojstaczer S., Cohn D., Marom G. (1985). Thermal-Expansion of Kevlar Fibers and Composites. J. Mater. Sci. Lett..

[65] (2023). CST Aramid (Kevlar^®^) Yarn Data. https://www.cstsales.com/kevlar_yarn_data.html.

[66] DuPont (2017). Kevlar^®^ Aramid Fiber Technical Guide. https://www.dupont.com/content/dam/dupont/amer/us/en/safety/public/documents/en/Kevlar_Technical_Guide_0319.pdf.

[67] MatWeb Arlon Electronic Materials 45NK Woven Kevlar^®^ Reinforced Laminate and Prepreg. https://www.matweb.com/search/DataSheet.aspx?MatGUID=accc6aafb7cf435a8a50ebfeb7a29c88&ckck=1.

[68] Fakirov S.K. (1990). Reorganization processes proceeding in a microcalorimeter (How reliable is the DSC method?). Polym. Sci. U.S.S.R..

[69] Murthy N.S., Curran S.A., Aharoni S.M., Minor H. (1991). Premelting Crystalline Relaxations and Phase-Transitions in Nylon-6 and 6,6. Macromolecules.

[70] Ramesh C., Gowd E.B. (2001). High-temperature X-ray diffraction studies on the crystalline transitions in the α- and γ-forms of nylon-6. Macromolecules.

[71] Pascual-González C., Iragi M., Fernández A., Fernández-Blázquez J.P., Aretxabaleta L., Lopes C.S. (2020). An approach to analyse the factors behind the micromechanical response of 3D-printed composites. Compos. Part B Eng..

[72] Yang C.C., Tian X.Y., Li D.C., Cao Y., Zhao F., Shi C.Q. (2017). Influence of thermal processing conditions in 3D printing on the crystallinity and mechanical properties of PEEK material. J. Mater. Process. Technol..

[73] Millot C., Fillot L., Lame O., Sotta P. (2015). Assessment of polyamide-6 crystallinity by DSC: Temperature dependence of the melting enthalpy. J. Therm. Anal. Calorim..

[74] Awaja F. (2016). Autohesion of polymers. Polymer.

[75] Yebra-Rodríguez A., Alvarez-Lloret P., Cardell C., Rodríguez-Navarro A.B. (2009). Crystalline properties of injection molded polyamide-6 and polyamide-6/montmorillonite nanocomposites. Appl. Clay Sci..

[76] Ito M., Mizuochi K., Kanamoto T. (1998). Effects of crystalline forms on the deformation behaviour of nylon-6. Polymer.

[77] Liu Y., Cui L., Guan F., Gao Y., Hedin N.E., Zhu L., Fong H. (2007). Crystalline Morphology and Polymorphic Phase Transitions in Electrospun Nylon 6 Nanofibers. Macromolecules.

[78] Xu J., Ren X., Yang T., Jiang X., Chang W., Yang S., Stroeks A., Chen E. (2018). Revisiting the Thermal Transition of β-Form Polyamide-6: Evolution of Structure and Morphology in Uniaxially Stretched Films. Macromolecules.

[79] Salvi A., Marzullo F., Ostrowska M., Dotelli G. (2025). Thermal Degradation of Glass Fibre-Reinforced Polyamide 6,6 Composites: Investigation by Accelerated Thermal Ageing. Polymers.

[80] Liao D., Gu T., Yan J., Yu Z., Dou J., Liu J., Zhao F., Wang J. (2024). Effect of thermal aging on the microscale mechanical response behavior of glass fiber/epoxy composites. J. Mater. Sci..

[81] Guo M., Liu G., Liao S. (2021). Normalized techno-economic index for renewable energy system assessment. Int. J. Electr. Power Energy Syst..

